# *De Novo* Trisomy 1q10q23.3 Mosaicism Causes Microcephaly, Severe Developmental Delay, and Facial Dysmorphic Features but No Cardiac Anomalies

**DOI:** 10.1155/2016/2861653

**Published:** 2016-01-31

**Authors:** Shirley Lo-A-Njoe, Lars T. van der Veken, Clementien Vermont, Louise Rafael-Croes, Vincent Keizer, Ron Hochstenbach, Nine Knoers, Mieke M. van Haelst

**Affiliations:** ^1^Department of Pediatrics, Horacio Oduber Hospital, Oranjestad, Aruba; ^2^Department of Genetics, Wilhelmina Children's Hospital, UMC Utrecht, 3584 EA Utrecht, Netherlands

## Abstract

Proximal duplications of chromosome 1q are rare chromosomal abnormalities. Most patients with this condition present with neurological, urogenital, and congenital heart disease and short life expectancy. Mosaicism for trisomy 1q10q23.3 has only been reported once in the literature. Here we discuss a second case: a girl with a postnatal diagnosis of a* de novo* pure mosaic trisomy 1q1023.3 who has no urogenital or cardiac anomalies.

## 1. Introduction

Proximal duplications of chromosome 1q are rare chromosomal abnormalities. Affected patients present with neurological, urogenital, and congenital heart anomalies as reported by Chen et al. [1], Mertens et al. [2], Machlitt et al. [3], Patel et al. [4], and Sifakis et al. [5]. Mosaicism for 1q10q23.3 duplication has only been reported once by Hirshfeld et al. [6]. Here, we report a second case with a postnatal diagnosis of a* de novo* pure mosaic trisomy 1q10q23.3. Although the girl has a developmental delay and similar facial dysmorphism as the previous reported case, she has no cardiac or urogenital anomalies. The absence of cardiac and urogenital anomalies is of importance for prognosis and illustrates the importance of (prenatal) counseling of parents of patients with trisomy 1q10q23.3 mosaicism.

## 2. Case Report

**Figure 1:**
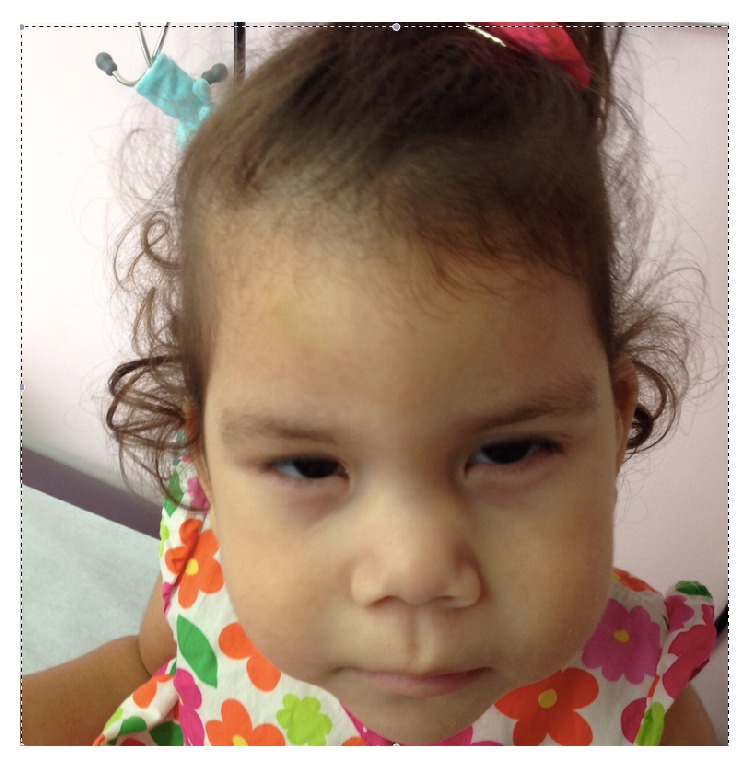
Face: note microcephaly; metopic ridge; wide, depressed nasal bridge; long philtrum; full cheeks; and retrognathia.

The proband was the first child of healthy unrelated Caribbean parents. She was born after a term pregnancy with a birth weight of 3120 g. The mother was 19 years old and the father was 22 years old at time of delivery. Apart from her father's sickle cell disease (HbSC), family history is noncontributory. Pregnancy care was performed by a midwife. Because of the uncomplicated pregnancy and the fact that Aruba does not yet have standardized 20-week fetal screening, no additional fetal testing (fetal ultrasound or prenatal genetic testing) was performed. The neonatal period was uncomplicated. At nine months of age she was referred to the pediatrician for evaluation of developmental delay. At that time she had a variable head lag and could not turn or sit independently. Feeding was uneventful and no illnesses, medication, or admissions were noted. On clinical examination, length was 71 cm (75th percentile), weight was 7650 g (10th percentile), and head circumference was 42 cm (<3th percentile). She was microcephalic and had a metopic ridge, small palpebral fissures with epicanthic folds, two naevi on the right side of the face, a wide depressed nasal bridge, a full and long philtrum, retrognathia, creases in the earlobes, a narrow palate, full cheeks, dimples on both elbows, rocker bottom feet, hemangioma on left hallux, and a sacral dimple (Figure 1). Neurological examination revealed axial hypotonia and variable head lag. Additional testing was performed from June 2013 till June 2014. Echocardiography showed a structural and functional normal heart, with a normal aortic arch; there were no signs of (hypertrophic) cardiomyopathy. Electrocardiography showed a normal sinus rhythm, sinus arrhythmia, no preexcitation, and no signs of ventricle hypertrophy, a normal conduction and repolarization. A CT of the brain showed a normal aspect of corpus callosum and normal basal ganglia, and there were no calcifications. MRI of the brain showed subendymal heterotopia towards the right lateral ventricle, without other abnormalities. Sonography of the kidneys showed normal kidneys and urinary tract. Ultrasound of the spine suggested spina bifida; however, MRI of the spine showed only lumbarization of S1 without signs of spina bifida.

Biochemical investigations in blood showed, apart from HbSC, no hematologic, renal, or liver function abnormalities. Cytogenetic analyses on blood lymphocytes showed a mosaic duplication of arm of chromosome 1q10 to 1q23.3. Because the proband was delivered by a midwife after an uneventful pregnancy and delivery she was not seen by a physician, as such no testing was performed at birth of child or placenta. Parental karyotypes were normal.

**Figure 2:**
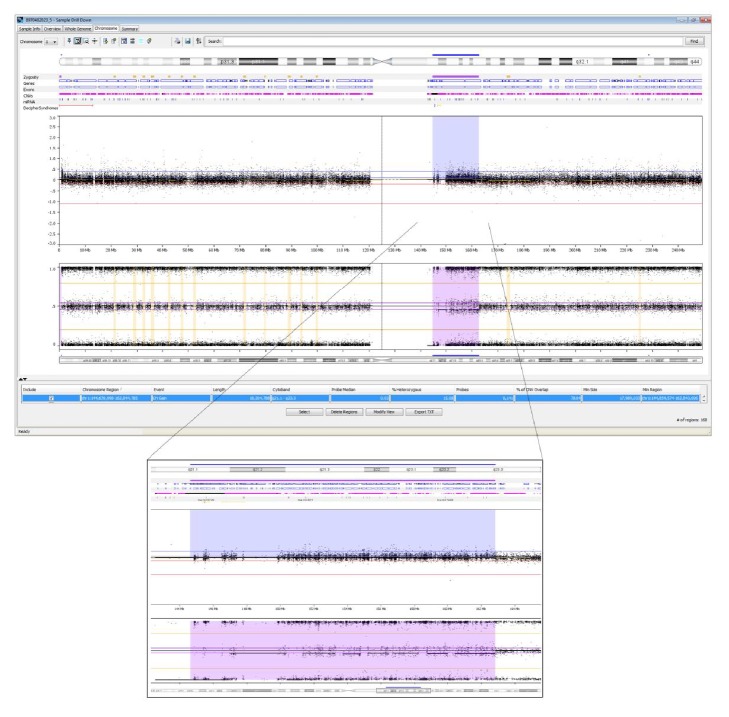
SNP-array analysis showed a mosaic duplication of ~18.0 Mb in 1q21.1-1q23.3: arr[hg19] 1q21.1q23.3(144,854,574-162,843,606) × 2~3. The upper *y*-axis shows the Log2 R ratio and the lower *y*-axis indicates the B allele frequency.

SNP-array analysis showed a female array profile suggestive of a mosaic copy number gain of ~18.0 Mb in chromosomal region 1q21.1q23.3 (8141 probes) (Figure 2): ISCN 2013 nomenclature, ISCN [7]: arr[hg19] 1q21.1q23.3(144,854,574-162,843,606) × 2~3.

**Figure 3:**
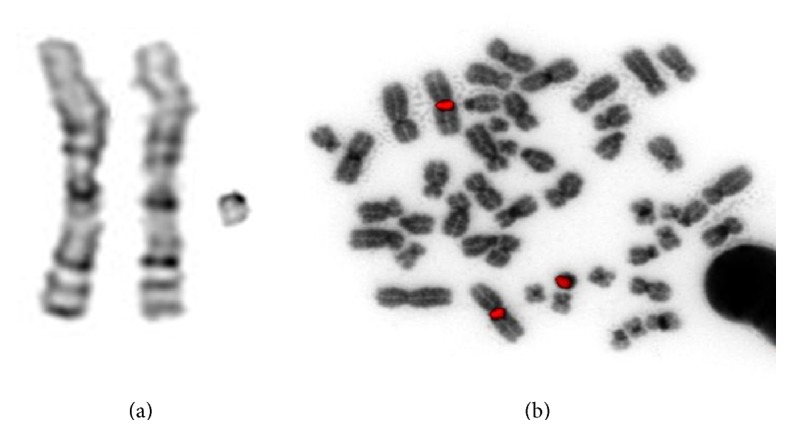
Partial G-banded karyogram showing both normal chromosomes 1 and the supernumerary der(1)(:q10→q23.3:) (a) and a metaphase after FISH using a satellite III DNA-probe (Vysis) showing three signals on band 1q12 (b).

Routine karyotyping confirmed the mosaic copy number gain as suspected by the SNP-array investigation. A supernumerary derivative of chromosome 1 was detected in 4 out of 16 metaphases; analyzing the remaining metaphases showed a normal female karyotype. The derivative consisted of the proximal part of the long arm of chromosome 1 from the centromere to the breakpoint in band 1q23.3 (Figure 3). Follow-up investigation in both parents indicated that the duplication arose* de novo.* The parental origin of the duplicated region was not investigated. The mosaic nature of the duplication probably indicates that the duplication was generated during one of the early postzygotic mitoses.

Thus far there is only one case reported in the literature with mosaicism for overlapping partial trisomy 1q duplication Hirshfeld et al. [6]. This previously reported patient had similar facial dysmorphic features to the present patient (Figure 1). In addition, she had mild bilateral hydronephrosis and hypertrophic cardiomyopathy with left ventricular outflow tract obstruction and Wolff-Parkinson-White syndrome.

Other presented cases with proximal trisomy 1q duplications presented with a wide range of neurological, urogenital, and congenital heart anomalies (Table 1). The authors hypothesize that the cardiac anomalies could be caused by a disruption or increased dosage effect of* TPM3* which is located within the duplicated region. Since cardiac anomalies are absent in the present case and her duplicated chromosomal region harbors (apart from* LMXA1*) the same genes, it is less likely that* TPM3* is a candidate gene for dilated and hypertrophic cardiomyopathy. The cardiac phenotype could thus result from mutations in other sarcomeric genes. The percentage of mosaicism for 1q10q23.3 duplication in the heart can however not be predicted from the performed analysis in blood and could completely differ between the present and previously reported case, possibly explaining the absence of cardiac anomalies in our case. An increased dosage effect of candidate gene* TPM3* could then still result in congenital heart disease in the previous case. Although we did not reveal cardiac abnormalities at the age of 1 year in the present case, a spontaneous closed foramen ovale or patent ductus arteriosus or transient hypertrophy in our case cannot be fully excluded. As yet, no rhythm or conduction disturbances have been noted, but follow-up will occur yearly.

In conclusion, this report suggests that congenital heart disease and urogenital abnormalities are no clear diagnostic features of mosaic trisomy 1q10q23.3. However, as with all mosaic chromosomal anomalies, the severity of affected organs is difficult to predict as it depends on the percentage of mosaicism of the chromosomal abnormality in the specific organ systems. Careful genetic counseling is warranted in case of (prenatal) detection of pure* de novo* trisomy 1q10q23.3 duplication as well as regular cardiac follow-up screening for similar cases without cardiac anomalies. Since this is only the second report of a mosaic trisomy 1q10q23.3, further cases need to be reported to further delineate the associated phenotype.

## Figures and Tables

**Table 1:** Clinical features in 7 patients, including presented case, with duplication of the proximal long arm of chromosome 1.

	Mertens et al., 1987 [2]	Chen et al., 2008 [1]	Hirshfeld et al., 2001 [6]	Machlitt et al., 2005 [3]	Patel et al., 2009 [4]	Sifakis et al., 2014 [5]	Present case
Karyotype	46,XY,inv dup(1)(q11→q22)	46,XY,dir dup(1)(pter→q25::q12→qpter)	46,XX,dir dup(1)(pter→q23::q12→q23::q23→qter)/46 XX	46,XY,der(1)(1qter→ q21::1p36.3→qter)	46,XY,+1,der(1;22)(q10;q10) [25]/46,XY[65]	46,XX,der(1)(pter→q31::q31→q12::q31→qter)	47,XX,+der(1)(::q10→q23.3::)[4]/46,XX[12].ish der(1)(CEP1+,wcp1+)

% mosaic				Amniotic fluid, 100%	27%	100%	Blood, 25%

Age of Dx	Delivery	Delivery	Delivery	Prenatal	Postmortem	Prenatal	9 months

GA (wk)	37	Term	Term	23	39	22,4	Term

BW (g/percentile)	2800/P25	3260/P25	3100/P25	440/P12	3300/P25	501/P12	3120/P25

Sex		Male	Female	Male	Male	Female	Female

Skull anomalies	+	+	−	+	−	−	+

Brain anomalies	+	+	+	+	+	+	−

Abnormal palate	+	+	+	u	+	−	−

Micro/retrognathia	+	+	+	+	+	+	+

Low set/malrotated ears	+	+	+	+	+	+	−

Eye anomalies	−	−	−	+	u	−	−

Cardiovascular anomalies		+	+	+		−	−

Respiratory anomalies		+	−	−		−	−

Gastrointestinal anomalies	+	+	+	+		+	−

Kidney anomalies	−	−	+	+		+	−

Genital anomalies	−	+	−	+		−	−

Hand/foot anomalies	+	+	+	+		+	+

Others	Excessive neck skin		Selective deficiency antibody response to polysaccharide antigens	SUA		13 pair ribs, defect vertebra bodies, and collagenopathy	HbSC

Survival	11 months	Dead 2 weeks	15 yr	TOP		TOP	2 years

Dx: diagnosis; GA: gestational age; BW: birth weight; SUA: single umbilical artery; HbSC: sickle cell type SC; TOP: termination of pregnancy; +: present; −: absent; u: unknown.
